# Thyrotoxic Crisis and COVID-19 Infection: An Extraordinary Case and Literature Review

**DOI:** 10.7759/cureus.11305

**Published:** 2020-11-02

**Authors:** Silvia Pastor, Ángeles Molina, Elena De Celis

**Affiliations:** 1 Neurology Department, La Paz University Hospital, Madrid, ESP; 2 Emergency Department, La Paz University Hospital, Madrid, ESP

**Keywords:** thyrotoxic, crisis, thyroid, disease, graves-basedow, covid-19, sars-cov-2, emergency

## Abstract

The novel coronavirus SARS-CoV-2, which causes the disease commonly known as COVID-19, has spread around the world, associated mostly with respiratory tract symptoms. We report the first case of a thyrotoxic crisis precipitated by COVID-19 and describe its identification, diagnosis, and management in the emergency unit. We also conduct a systematic review of thyrotoxic crisis literature and COVID-19 infection. This case highlights the importance of considering the SARS-CoV2 virus as a potential trigger of a thyroid storm. It also shows the need to maintain extreme contact precautions even after one month of COVID-19 symptom onset.

## Introduction

SARS-CoV-2 is a novel coronavirus recently detected and responsible for the current global pandemic that began in December 2019 in Wuhan, China [1], and has rapidly spread throughout the world. The first case confirmed in Spain was reported on January 31, 2020, when a German tourist tested positive for SARS-CoV-2 in La Gomera, Canary Islands. The first case in the Madrid region was declared on February 27, 2020 [2]. Although most patients have respiratory tract symptoms, a wide range of symptoms attributable to the infection have been described, such as gastrointestinal [3] and neurological [4] symptoms, among others. The pathogenesis of COVID-19 uses angiotensin-converting enzyme 2 (ACE2) as a receptor. As a matter of fact, several endocrine organs do express ACE2, namely, the pancreas, thyroid, testis, ovary, adrenal glands, and pituitary [5]. For that reason, one could expect endocrine repercussions due to the interaction of SARS-CoV-2 with ACE2 expressed on these organs [6]. We describe the first case of thyrotoxic crisis in a patient with COVID-19 infection for more than one month. We also conducted a systematic review (SR) of the published literature regarding the effect of COVID-19 infection as a trigger of a thyrotoxic crisis.

## Case presentation

**Figure 1:**
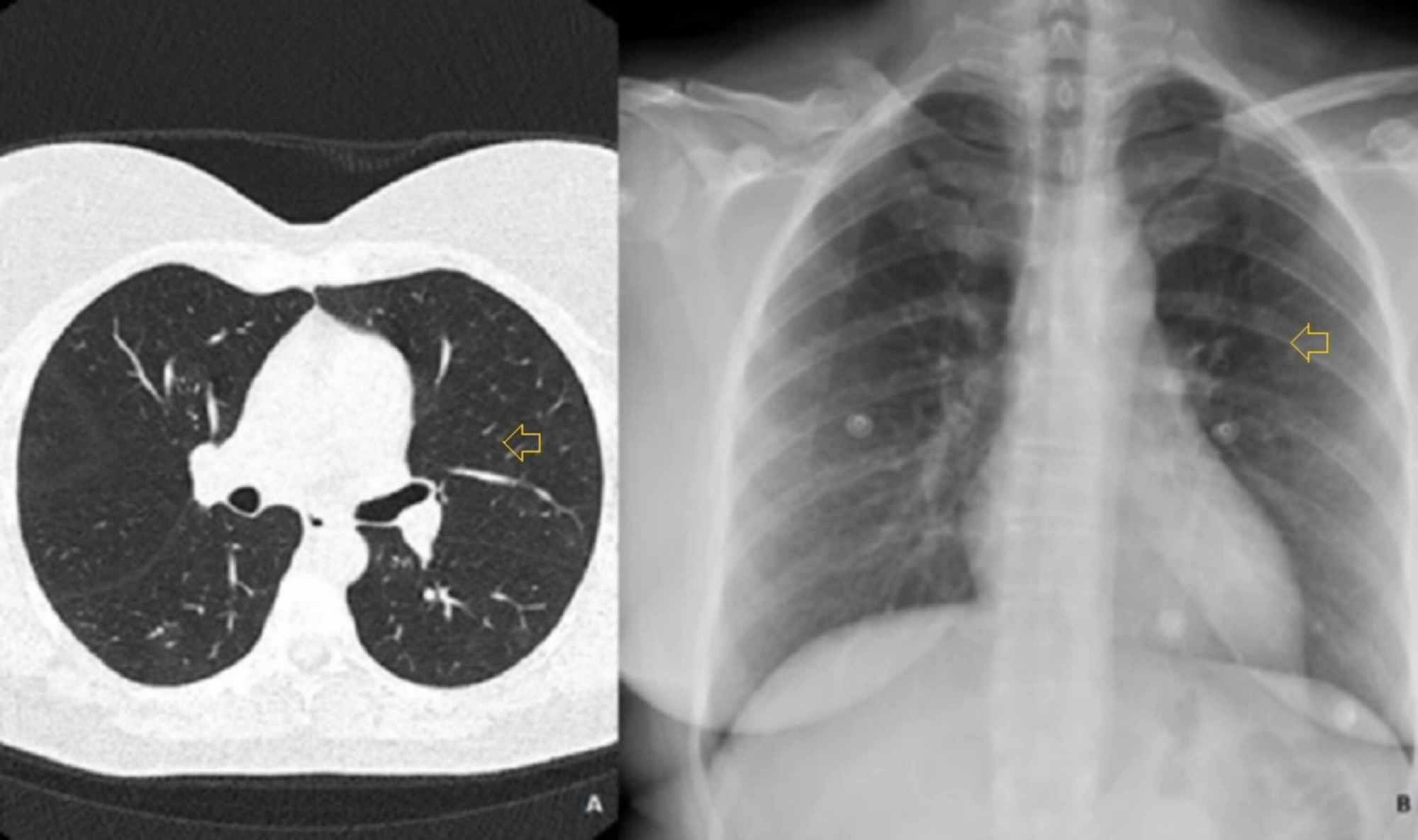
a. CT chest scan, b. Posteroanterior chest radiography No thoracic abnormalities were noted.

A 45-year-old Caucasian woman, with a previous history of Graves’ disease in stable remission for more than four years without any medical or surgical treatment, presented to the emergency department (ED) with a two-day history of anxiety and palpitations. She had previously attended the ED for presenting fever, cough, sore throat, and certain difficulty breathing 38 days before. A computed tomography (CT) chest scan (Figure 1a) was performed on that occasion, with normal results. No SARS-CoV-2 reverse transcription-polymerase chain reaction (RT-PCR) was conducted, although she was discharged with a diagnosis of possible COVID-19 infection and was given symptomatic treatment. She experienced a 4 kg weight loss and irregular menses the following month, with the cessation of respiratory symptoms two weeks before attending the ED again. On this occasion, she described sleep difficulty, excessive sweating, and heat intolerance for the last 48 hours. She denied any symptoms related to an acute infection, personal history of diabetes, any history of recent surgery or trauma, radioactive iodine treatment or exposure to iodinated contrast, withdrawal of antithyroid treatment, or the possibility of being pregnant, nor beginning any treatment. On ED presentation, she had 37.3ºC body temperature and sinus rhythmic tachycardia at 180 bpm. Her blood pressure was stable at 133/89 mmHg. On examination, the patient was agitated and anxious although she did not show signs of altered consciousness (Glasgow Coma Scale (GCS) 15). She was diaphoretic, flushed, and had bilateral ocular proptosis and conjunctival hyperemia on examination. Neither jugular venous distention nor peripheral edema was observed. She had regular tachycardia on otherwise normal cardiac and pulmonary auscultation. No thyromegaly was observed on physical examination nor any other abnormalities. A chest X-ray (Figure 1b) was also performed, with unremarkable findings.

Initial complete blood count, basic metabolic panel (Table 1), and urinalysis were all normal. We only observed slightly increased values of fibrinogen, D-dimer, and lactose dehydrogenase (LDH). The urine pregnancy test was negative. The patient’s thyroid function tests on the day of presentation were thyroid-stimulating hormone <0.01 µU/mL (normal values 0.55-4.78) and free thyroxine 3.29 ng/dL (normal values 0.89-1.76). An electrocardiogram (ECG) showed sinus tachycardia. Although the patient reported respiratory symptoms more than one month before, in the absence of a clear trigger for a thyrotoxic crisis, we requested an RT-PCR assay from the nasopharyngeal swab, with positive results.

**Table 1:** Clinical laboratory results

Measure	Reference Range	Results
White-cell count (per μl)	3,900-10,200	8,350
Red-cell count (per μl)	3,900-5,200	4,360
Absolute neutrophil count (per μl)	1,500-7,700	4,130
Absolute lymphocyte count (per μl)	11,00-4,500	2,890
Platelet count (per μl)	150,000-370,000	366,000
Hemoglobin (g/dl)	12.0-15.6	12.9
Hematocrit (%)	35.5-45.5	39.8
Sodium (mmol/liter)	136-145	142
Potassium (mmol/liter)	3.5-5.1	3.7
Chloride (mmol/liter)	99-109	109
Glucose (mg/dl)	74-106	96
Creatinine (mg/dl)	0.50-1.10	0.63
Total protein (g/dl)	6.4-8.3	7.4
Albumin (g/dl)	2.9-5.2	4.5
Total bilirubin (mg/dl)	0.30-1.20	0.37
Alanine aminotransferase (U/liter)	<40	<8
Aspartate aminotransferase (U/liter)	<35	31
Lactate dehydrogenase (U/liter)	100-190	239
Gamma-glutamyl transpeptidase (U/liter)	<38	29
Troponine I (ng/ml)	<34.1	18.7
Creatine kinase (U/liter)	35-210	117
C-reactive protein (mg/liter)	0.0-5.0	4.5
D-dimer (ng/ml)	0-500	670
Fibrinogen (md/dl)	150-450	506
Prothrombin time (sec)	70-120	11.8

According to the Burch-Wartofsky Point Scale (Appendix 1), the patient achieved 50 points, highly suggestive of thyroid storm.

A regimen of atenolol 50 mg every 12 hours was initiated in order to achieve heart rate control; paracetamol and hydrocortisone 100 mg every eight hours were also administered. The patient was also started on thiamazole with an initial loading dose of 30 mg followed by 30 mg every six hours. Intensive care management was finally unnecessary because of the patient´s good response, achieving normal heart rate (100 bpm) and no central nervous system symptoms within the following hours.

## Discussion

In addition to presenting a clinical case, we conducted a systematic review (April 26, 2020- May 1, 2020). Two reviewers (SP and AM) independently searched PubMed and Embase using different search strategies (Appendices 2 and 3) including a combination of relevant terms focused on cases with COVID-19 and thyrotoxic crisis.

**Figure 2:**
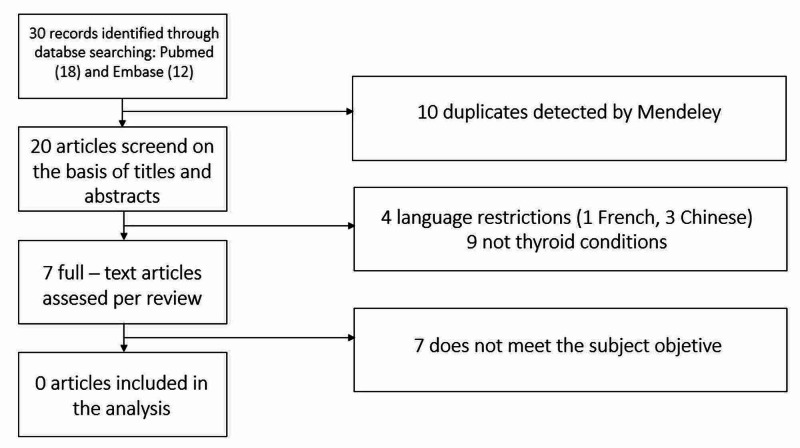
Flowchart

Thirty articles (18 PubMed, 12 Embase) were identified and exported to Mendeley software. Even though seven articles involved thyroid pathology, we did not find any case report of a thyrotoxic crisis triggered by COVID-19 infection. Figure 2 shows the flowchart for the systematic review.

A review of the currently available literature shows that this is the first case of a thyrotoxic crisis probably precipitated by COVID-19 infection.

In national researches from the United States and Japan, the incidence of thyroid storm was 0.57 to 0.76 and 0.20 per 100,000 persons per year, respectively [7-8]. Although thyroid storms may arise in patients with longstanding untreated hyperthyroidism, they are often precipitated by an acute underlying event. In the Swee series [9], non-compliance with treatment was a major trigger in previously hyperthyroid diagnosed patients, followed by infection.

We did not initially suspect an active COVID-19 infection since the patient reported the onset of symptoms more than 30 days before attending the ED with anxiety and tachycardia, and had no respiratory symptoms for more than 14 days. However, we must highlight that the duration of viral RNA shedding is variable. In the Xiao study [10], the median duration between the onset of symptom to nucleic acid conversion was 24 days and the longest was 42 days.

There is some evidence that the virus may have a certain tropism for the thyroid gland. ACE2 expression levels have recently been studied in 31 normal human tissue samples, detecting the highest in thyroid, testis, kidneys, heart, and adipose tissue, presuming that SARS-CoV-2 may infect other tissues aside from the lungs [11].

It has been described that during the SARS outbreak in 2003, a sick euthyroid syndrome was observed. However, there are no publications on the euthyroid sick syndrome in COVID-19 patients. In the Leow series [12], 6.7% out of 61 patients developed hypothyroidism after SARS infection (three with central hypothyroidism and one with primary hypothyroidism).

Currently, a new clinical trial (NCT04348513) is investigating the effect of high doses of intravenous T3 in severely ill patients with sepsis caused by COVID-19. This study is based on the presumption that viral sepsis produces the deregulation of thyroid hormones (TH) with low circulating T3 levels. As TH could increase the tolerance of the cell to hypoxia and potentiate host defense by increasing natural killer cells, treatment with intravenous T3 could enhance the recovery of critically ill patients [13].

The different epidemiological and clinical series of Huang [14], Wang [15], Guan [16], Goyal [17], and Chen [18] did not reflect any case of hyperthyroidism as comorbidity. The effect of COVID-19 in diabetic patients has been studied, as well as the need for the more intensive management of blood glucose levels, but nevertheless, there are important unresolved questions regarding the effect of the virus on the thyroid gland.

We found different case reports involving thyroid disease and COVID-19 infection, but neither of them is related to a thyroid storm:

- A 65-year-old woman with autoimmune hypothyroidism, hypertension, and SARS-CoV-2 exposure came to the ED with a four-day history of fatigue, fever, and dry cough. She also developed lower extremity purpura. Her level of thyroid peroxidase antibodies was 245 UI/ml, the platelet count on Day 5 was 16,000 per cubic millimeter and on Day 7, it was 8000, the prothrombin and activated partial thromboplastin times were normal, and a peripheral-blood smear showed less than 1% schistocytes. She finally was diagnosed with immune thrombocytopenic purpura. The authors suggested that COVID-19 was a causal factor in immune thrombocytopenia in this patient [19].

- An 18-year-old girl consulted for fever, neck pain, and palpitations occurring 15 days after a SARS-CoV-2 positive oropharyngeal swab. At the physical examination, she had a heart rate of 90 beats per minute and a painful and enlarged thyroid on palpation. At laboratory exams, T3 and T4 were high, thyrotropin undetectable, and inflammatory markers and white blood cell count elevated, with hypoechoic areas detected at neck ultrasound. She was diagnosed with subacute thyroiditis [20].

Evidence regarding thyroid function and thyroid pathology are not yet available in COVID-19. The cytokine response described in COVID-19 seems to resemble, at least in part, the immune activation that accompanies inflammatory thyroid diseases. Specifically, a hyperactivation of Th1 response in peripheral lymphocytes was described in patients with autoimmune and drug-induced thyroiditis, and an increase in IL-6 was reported in the course of destructive thyroiditis. As a matter of fact, alterations of thyroid function and structure may occur during COVID-19, and these could be as a consequence of direct or indirect effects of SARS-CoV-2 on the thyroid gland. The British Thyroid Association and the Society for Endocrinology have elaborated a consensus statement regarding issues specific to thyroid dysfunction during the COVID-19 pandemic, emphasizing that patients with uncontrolled thyroid disease (especially thyrotoxicosis) may be at a higher risk of complications like thyroid storm from any infection, and they are advised to continue their prescribed medications as usual to reduce this risk. There is a lack of information about the prevalence and determinants of thyrotoxicosis and COVID-19, and more studies are needed.

The American Thyroid Association has recommended a 90-day supply of prescriptions or receiving their thyroid medications through a mail-order service instead of picking them up at the local pharmacy in order to maintain social distancing and limit exposure to COVID-19.

## Conclusions

Our case of thyroid storm and concomitant COVID-19 infection suggests that SARS-CoV2 infection may be a trigger for the patient´s thyrotoxic crisis. This case report highlights the importance of clinicians eliciting a recent history of mild respiratory disease suspected to be COVID-19 to ensure appropriate identification and prompt isolation in order to reduce further transmission.

In addition, we recommend caution in cases of patients with a previous history of Graves’ disease, as SARS-CoV-2 could provoke a thyrotoxic crisis as occurred in the case we report. More studies are needed in order to propose recommendations for the early diagnosis and management of patients with Graves' disease and COVID-19 infection who could evolve to a thyrotoxic storm, a rare but potentially fatal condition.

## Appendices

Appendix 1 

**Table 2:** Burch-Wartofsky Point Scale

Criteria	Points
Temperature (ºC)	
37.2-37.7	5
37.8-38.3	10
38.4-38.8	15
38.9-39.4	20
39.4-39.9	25
≥40.0	30
Tachycardia (beats per minute)	
100-109	5
110-119	10
120-129	15
130-139	20
≥140	25
Atrial fibrillation	
Absent	0
Present	10
Congestive heart failure	
Absent	0
Mild	5
Moderate	10
Severe	20
Gastrointestinal-hepatic dysfunction	
Absent	0
Moderate	10
Severe	15
Central nervous system disturbance	
Absent	0
Mild	10
Moderate	20
Severe	30
Precipitating event	
Absent	0
Present	10

Appendix 2

PubMed Search Strategy

((sars-cov2) OR (sarscov2) OR (covid) OR (covid19)) AND ((thyroid) OR (thyroid disease) OR (hyperthyroidism) OR (thyrotoxicosis) OR (thyroid storm) OR (thyrotoxic crisis))

Appendix 3

Embase Search Strategy

#12 #10 OR #11

#11 #5 OR #6 OR #7 OR #8 OR #9

#10 #1 OR #2 OR #3 OR #4

#9'thyroid crisis'

#8'thyrotoxicosis'

#7'hyperthyroidism'

#6'thyroid AND disease'

#5'thyroid'

#4'covid19'

#3'covid'

#2'sarscov2'

#1'sars cov2'
